# Nutrient supply affects the mRNA expression profile of the porcine skeletal muscle

**DOI:** 10.1186/s12864-017-3986-x

**Published:** 2017-08-10

**Authors:** Tainã Figueiredo Cardoso, Raquel Quintanilla, Joan Tibau, Marta Gil, Emilio Mármol-Sánchez, Olga González-Rodríguez, Rayner González-Prendes, Marcel Amills

**Affiliations:** 1 Department of Animal Genetics, Center for Research in Agricultural Genomics (CSIC-IRTA-UAB-UB), Universitat Autònoma de Barcelona, 08193 Bellaterra, Spain; 2 CAPES Foundation, Ministry of Education of Brazil, Brasilia D. F., Zip Code 70.040-020 Brazil; 3 Animal Breeding and Genetics Program, Institute for Research and Technology in Food and Agriculture (IRTA), Torre Marimon, 08140 Caldes de Montbui, Spain; 4 IRTA-Monells, Finca Camps i Armet s/n 17121, Monells, Spain; 5 Departament de Ciència Animal i dels Aliments, Facultat de Veterinària, Universitat Autònoma de Barcelona, 08193 Bellaterra, Spain

**Keywords:** Pig, RNA-seq, Oxidative stress, Transcription factor, Circadian rhythm, Angiogenesis

## Abstract

**Background:**

The genetic basis of muscle fat deposition in pigs is not well known. So far, we have only identified a limited number of genes involved in the absorption, transport, storage and catabolism of lipids. Such information is crucial to interpret, from a biological perspective, the results of genome-wide association analyses for intramuscular fat content and composition traits. Herewith, we have investigated how the ingestion of food changes gene expression in the *gluteus medius* muscle of Duroc pigs.

**Results:**

By comparing the muscle mRNA expression of fasted pigs (T0) with that of pigs sampled 5 h (T1) and 7 h (T2) after food intake, we have detected differential expression (DE) for 148 (T0-T1), 520 (T0-T2) and 135 (T1-T2) genes (*q*-value <0.05 and a |FC| > of 1.5). Many of these DE genes were transcription factors, suggesting that we have detected the coordinated response of the skeletal muscle to nutrient supply. We also found DE genes with a dual role in oxidative stress and angiogenesis (*THBS1, THBS2* and *TXNIP*), two biological processes that are probably activated in the post-prandial state. Finally, we have identified several loci playing a key role in the modulation of circadian rhythms (*ARNTL, PER1, PER2, BHLHE40, NR1D1, SIK1, CIART* and *CRY2*), a result that indicates that the porcine muscle circadian clock is modulated by nutrition.

**Conclusion:**

We have shown that hundreds of genes change their expression in the porcine skeletal muscle in response to nutrient intake. Many of these loci do not have a known metabolic role, a result that suggests that our knowledge about the genetic basis of muscle energy homeostasis is still incomplete.

**Electronic supplementary material:**

The online version of this article (doi:10.1186/s12864-017-3986-x) contains supplementary material, which is available to authorized users.

## Background

Physiological genomics aims to understand the molecular basis of highly complex biological processes by applying high-throughput technologies to the large-scale analysis of genomes, transcriptomes and proteomes [1]. We have a very limited understanding of the physiological genomics of intramuscular fat (IMF) content and composition traits in pigs. Several RNA-seq studies comparing the muscle transcriptomes of pigs with divergent lipid profiles have been performed, demonstrating the differential expression of a number of genes related with carbohydrate and lipid metabolism [2–4]. Noteworthy, genome-wide association studies (GWAS) of blood lipid traits in humans have uncovered the existence of a large number of genes strongly associated with plasma lipid concentrations whose involvement in lipoprotein metabolism had never been reported before [5]. For instance, Teslovich et al. [6] performed a GWAS for lipid traits in 100,000 individuals and identified several associated loci (e.g. *GALNT2*, *PPP1R3B*, and *TTC39B*) whose participation in lipid metabolism had not been described previously. Similarly, the Global Lipids Genetics Consortium reported 62 novel loci displaying significant associations with blood lipid levels, and 30 of them had never been previously connected to lipid metabolism [7]. In the light of these results, we can infer that many genes contributing to muscle fat deposition remain to be identified.

The skeletal muscle compartment encompasses a substantial fraction of the body weight and accounts for ≈75% of total insulin-stimulated glucose uptake [8]. Moreover, adipose and muscle tissues absorb most of the chylomicrons generated after a meal consumption [9]. Fat deposition in the porcine muscle may depend, at least in part, on the activation of genes that regulate the uptake, transport, storage, synthesis and degradation of fatty acids (FA) and carbohydrates. As a first step to identify such genes, we have investigated how the profile of pig muscle mRNA expression changes in response to nutrient supply.

## Methods

### Animal material and metabolic profile

A group of 36 female piglets belonging to a commercial Duroc line were brought, after weaning (age = 3–4 weeks), to the IRTA-Pig Experimental Farm at Monells (Girona, Spain). They were fed with a transition feed for 40 days, and, at an approximate age of 2 months, they entered the fattening period. Gilts were housed individually and fed ad libitum with a commercial feeding diet (13% and 5.5% of crude protein and crude fat respectively) until they reached an average live weight of 73 ± 1.2 kg (161 ± 1.1 days). The post-prandial time-points at which muscle gene expression should be analysed were chosen on the basis of the following experiment (experiment 1): we selected at random eight Duroc gilts (out of the 36), with an approximate age of 100 days, and blood samples were taken with citrate Vacutainer tubes before feeding and 2, 4, 6 h. after feeding. These 32 samples were submitted to the Veterinary Clinical Biochemistry Service of the Universitat Autònoma de Barcelona (http://sct.uab.cat/sbcv). The following metabolites were measured using standard protocols: plasma glucose, triglycerides, cholesterol and non-esterified fatty acids.

In experiment 2, we analysed the transcriptomic changes associated with food intake by sequencing the muscle transcriptomes of the 36 Duroc gilts mentioned in the previous paragraph. These gilts were slaughtered at the IRTA-Experimental slaughterhouse in Monells (Girona, Spain) in controlled conditions and complying all national welfare regulations. These 36 sows fasted 12 h prior slaughtering and then 12 of them were stunned, with high concentrations of CO_2_ to minimize pain, and bled (T0, fasting). The remaining 24 gilts were supplied with a standard feed ad libitum, and slaughtered 5 h (T1, *N* = 12) and 7 h (T2, *N* = 12) after T0, following the same procedure reported above. Before slaughter, we took blood samples from these sows and triglyceride and plasma free FA were measured at the Veterinary Clinical Biochemistry Service of the Universitat Autònoma de Barcelona (http://sct.uab.cat/sbcv). After slaughtering, samples of the *gluteus medius* muscle were collected and submerged in RNAlater (Ambion), being stored at −80 °C until use.

### RNA isolation and library construction and sequencing

Each muscle sample was individually submerged in liquid nitrogen and pulverized with a mortar and a pestle. This powder was homogenized with a polytron device in 1 mL of TRI Reagent (Thermo Fisher Scientific, Barcelona, Spain). Total RNA was extracted from *gluteus medius* muscle samples by using the acid phenol method implemented in the RiboPure kit (Ambion, Austin, TX). Total RNA concentration and purity were assessed with a Nanodrop ND-1000 spectrophotometer (Thermo Fisher Scientific, Barcelona, Spain), while integrity was checked with a Bioanalyzer-2100 equipment (Agilent Technologies, Inc., Santa Clara, CA). Total RNA samples were submitted to the Centre Nacional d’Anàlisi Genòmica (CNAG, http://www.cnag.cat) for sequencing. Individual libraries for each one of the analysed pigs (*N* = 36) were prepared using the TruSeq Stranded mRNA Library Preparation Kit (Illumina Inc., CA) according to the protocols recommended by the manufacturer. This level of replication is 4-fold higher than the minimum required (3 individuals/group) in standard RNA-seq studies. Each library was paired-end sequenced (2 × 75 bp) in a HiSeq 2000 platform (Illumina Inc., CA) by using the TruSeq SBS Kit v3-HS (Illumina Inc., CA).

### Bioinformatic analyses

Quality control of sequence reads was carried out with the FASTQC software (Babraham Bioinformatics, http://www.bioinformatics.babraham.ac.uk/projects/fastqc/). We made per-sequence and per-base analyses to filter reads according to the following criteria: sequence-read distribution = 75 bp, 100% coverage in all bases, GC-content ~50%, ~25% of A, T, G and C nucleotide contributions, ambiguous base-content <0.1% and a Phred score higher than 30 (i.e. base-calling accuracy larger than 99.9%). Subsequently, sequences were trimmed for any remaining sequencing adapter by using Trimmomatic v.0.22 [10]. Raw reads were mapped to the pig reference genome (version 10.2-) with the *STAR* Alignment v.2.5. software [11] by using default parameters and *STAR 2*-*pass* alignment steps. The *FeatureCounts* tool [12] was used to summarize counts of unambiguously mapped reads. The expression of each mRNA was estimated with *DESeq2* [13]. This software builds a count matrix *K*_*ij*_ (with one row for each gene *i* and one column for each sample *j*) encompassing the number of sequencing reads that have been unambiguously mapped to a gene in a sample [13]. The main assumption of this method is that read counts follow a negative binomial distribution with mean μ_ij_ and dispersion *α*_*i*_ [13]. A second important assumption is that genes of similar average expression levels are expected to have a similar dispersion *α*_*i*_ value. *DeSeq2* calculates final dispersion values by using an empirical Bayes approach that shrinks dispersion estimates towards a set of predicted *α*_*i*_ values. When dealing with genes that are poorly expressed, log_2_ fold-change (FC) estimates can have a high variance due to noisiness issues. To avoid this potential problem, *DeSeq2* shrinks log_2_ fold-change estimates, with an empirical Bayes procedure [13]. Finally, a Wald test is used to infer if shrunken log_2_ fold-change estimates (and their standard errors) are significantly different from zero. In the Wald test, the shrunken estimate of the log_2_ fold-change is divided by its standard error, generating a *z*-statistic that can be compared to a standard normal distribution [13]. Correction for multiple testing is achieved by using a false discovery rate approach [14]. We considered as differentially expressed (DE) those mRNAs displaying a |FC| > 1.5 and a *q*-value <0.05.

Advaita Bio’s iPathwayGuide (http://www.advaitabio.com/ipathwayguide) and the Cytoscape software [15] combined with the ReactomeFIViz app [16] were used to infer if certain gene ontology terms and pathways are enriched across the sets of DE genes as well as to build biological networks. In order to detect the GO categories that are over- or under-represented in the condition under study, Advaita Bio’s iPathwayGuide uses an impact analysis method that relies on classical statistics but also takes into account other key factors such as the magnitude of each gene’s expression change, their type and position in the given pathways, their interactions, etc. [17]. The ReactomeFIViz application can access the Reactome pathways database in order to do pathway enrichment analysis for a set of genes and visualize hit pathways with the aid of Cytoscape [16]. This application can also access the Reactome Functional Interaction (FI) network to construct a FI sub-network based on a set of genes [16]. In our study, the standard ReactomeFIViz “Gene Set/Mutation Analysis” application was employed to build gene functional interaction networks on the basis of a list of DE genes (*q*-value <0.05 and a |FC| > of 1.5) and curated pathway information contained in the Reactome database. The functional enrichment analyses for pathways and GO annotations were based on a binomial test [16].

## Results

**Fig. 1:**
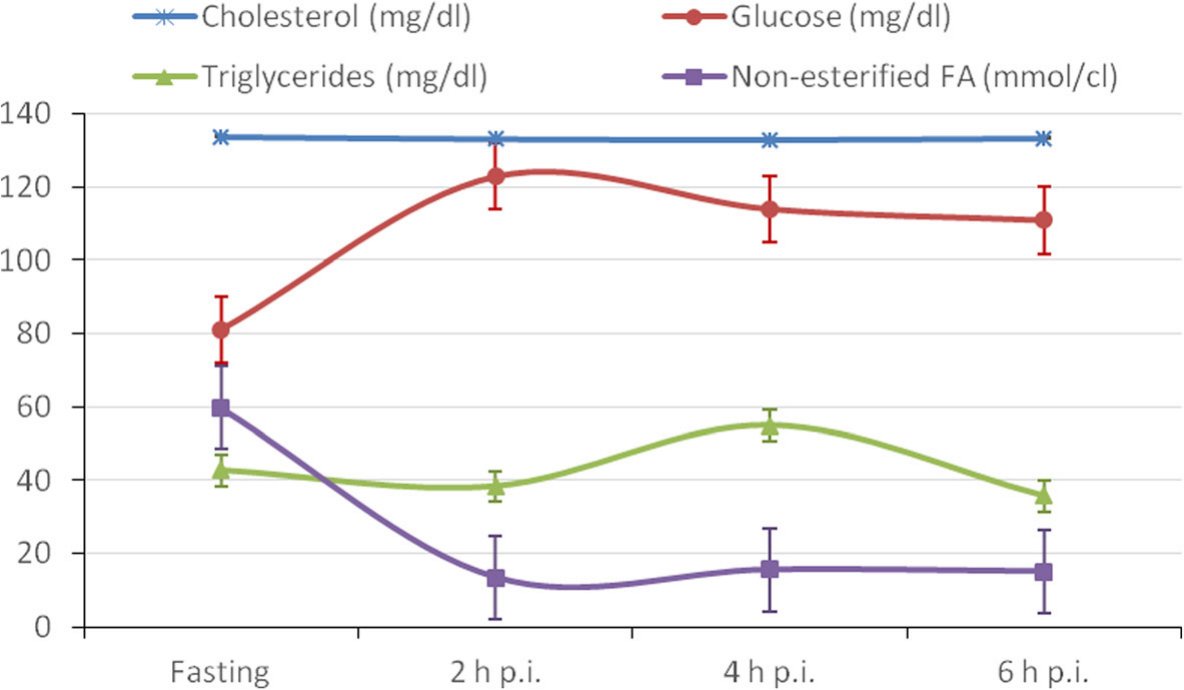
Kinetics of the average concentrations of plasma glucose, cholesterol, triglycerides and non-esterified fatty acids (FA) in 8 Duroc pigs at four time points: before eating and 2, 4 and 6 h post-ingestion (p.i)

In **Experiment 1**, measurement of the concentrations of plasma glucose, cholesterol, triglycerides and non-esterified fatty acids revealed that glycaemia and lipidemia peaks took place 2 and 4 h after the 8 Duroc gilts began to eat, a result that was very consistent across individuals (Fig. 1). Eating was also accompanied by a marked decrease of plasma free FA (Fig. 1), a finding that agrees well with the role of these metabolites as a source of energy during fasting. We chose 5 and 7 h post-ingestion as time-points to carry out the analysis of differential expression. Our expectation was that T1 would reflect the process of lipid absorption, while T2 would correspond to a posterior phase in which lipids are stored as triglycerides or catabolized in the β-oxidation pathway to generate ATP. Nevertheless, when we measured the concentrations of triglycerides and plasma free fatty acids in the slaughtered sows forming part of **Experiment 2** (Additional file 1: Figure S1), we observed that feeding is associated with an increase in the concentration of triglycerides and a decrease of circulating free FA levels, a result that matches the metabolic profile observed in **Experiment 1**. However, the kinetics of these two metabolites were not identical to those observed in **Experiment 1** because 7 h after feeding triglyceride levels were still peaking. Despite this circumstance, our main comparison (fasting vs fed pigs) remains completely valid.

**Fig. 2:**
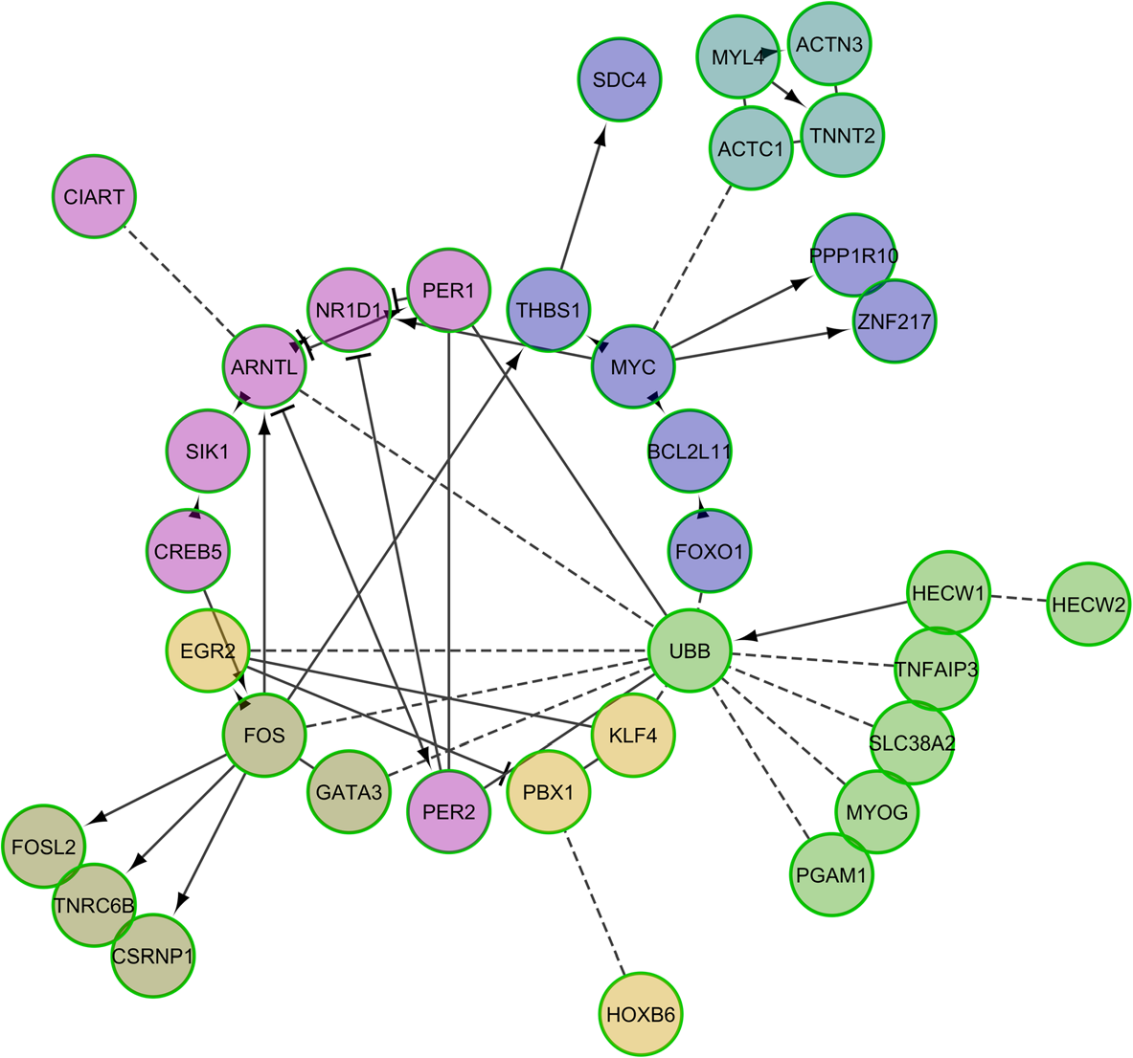
Reactome functional interaction network corresponding to 148 genes that show differential expression in the T0 (fasting) vs T1 (5 h after eating) comparison. Nodes in different network modules are displayed in different colors. Letters in parentheses represent the source database as follows: R – Reactome, K – KEGG, and B – BioCarta. Enriched pathways (*q*-value <0.05) in each one of the individual network modules are: 1: Proteoglycans in cancer (K); 2: TNF signaling (R); 3: Circadian clock (R); 4: Bone remodeling (B); 5: Striated muscle contraction (R) and 6: Transcriptional regulation of pluripotent stem cells (R)

**Fig. 3:**
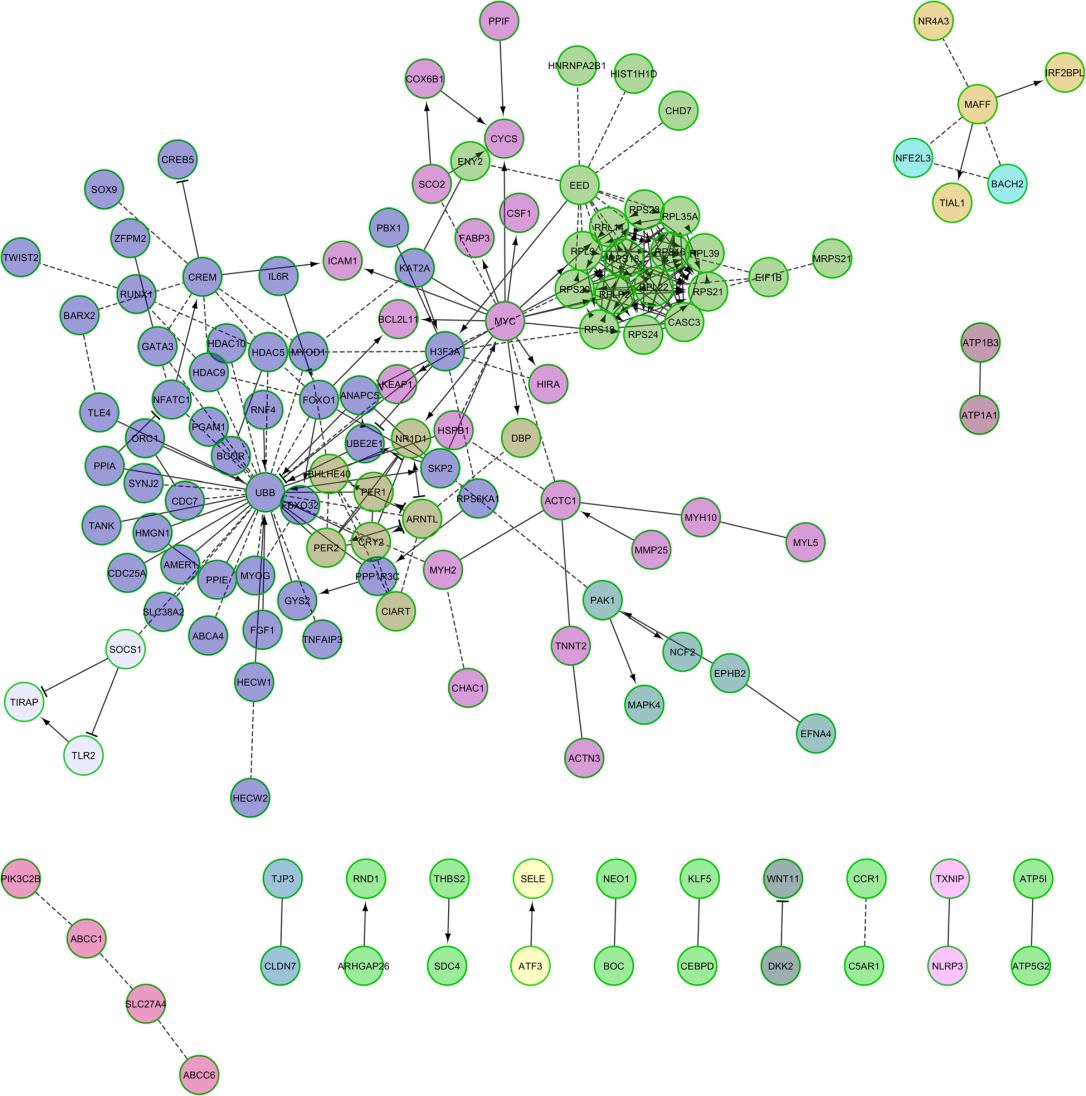
Reactome functional interaction network corresponding to 520 genes showing differential expression in the T0 (fasting) vs T2 (7 h after eating) comparisons. Nodes in different network modules are displayed in different colors. Letters in parentheses represent the source database as follows: R – Reactome, K – KEGG, N – NCI PID, P - Panther, and B – BioCarta. Enriched pathways (*q*-value <0.05) in each one of the individual network modules are: 1: Mitotic G1-G1/S phases (R); 2: Nicotinic acetylcholine receptor signaling pathway (P); 3: SRP-dependent co-translational protein targeting to membrane (R); 4: Senescence-associated secretory phenotype (SASP) (R); 5: Signaling events mediated by HDAC Class II (N); 6: Circadian rhythm pathway (N), 7: Oxidative stress induced gene expression via *Nrf2* (B); 8: ABC-family proteins mediated transport (R); 9: Toll-like receptors cascades (R); 11: Proximal tubule bicarbonate reclamation (K); 12: Wnt signaling pathway (K); 13: Nucleotide-binding domain, leucine rich repeat containing receptor (NLR) signaling pathways (R); 14: ATF-2 transcription factor network (N); 15: ECM-receptor interaction (K); 16: GPCR ligand binding (R); 17: Oxidative phosphorylation (K); 18: Integrin signalling pathway (P); 19: Myogenesis (R); 20: Transcriptional regulation of white adipocyte differentiation (R)

**Fig. 4:**
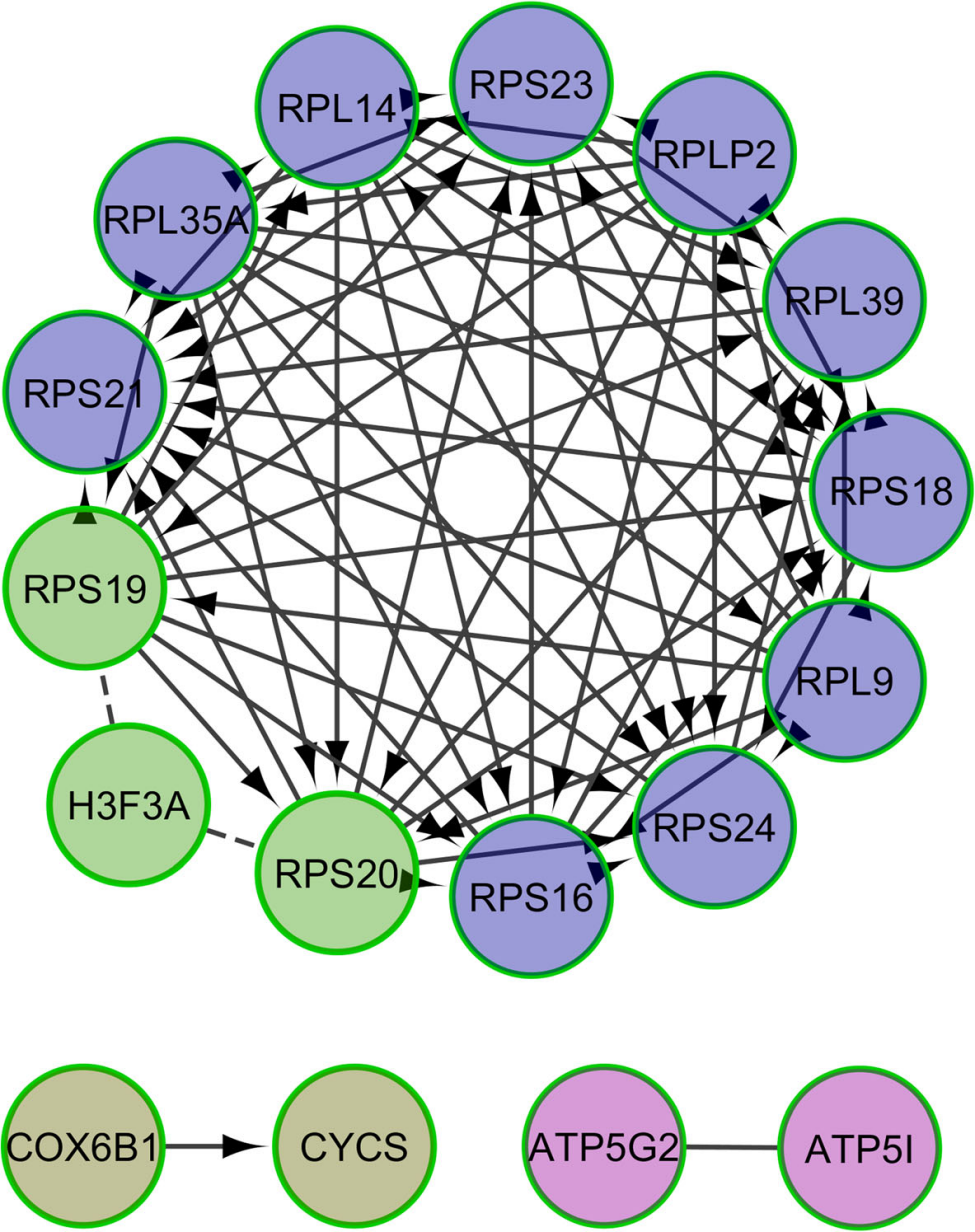
Reactome functional interaction network corresponding to 135 genes showing differential expression in the T1 (5 h after eating) vs T2 (7 h after eating) comparison. Nodes in different network modules are displayed in different colors. Letters in parentheses represent the source database as follows: R – Reactome and K – KEGG. Enriched pathways (*q*-value <0.05) in each one of the individual network modules are: 1: SRP-dependent cotranslational protein targeting to membrane (R); 2: Eukaryotic Translation Termination (R); 3: Oxidative phosphorylation (K) and 4: Parkinson’s disease (K)

The RNA-seq experiment generated an average of 45 million paired-end reads per sample and 69.8% of them were unambiguously mapped to the pig *Sscrofa10.2* genome assembly. Analysis of the data with *DESeq2* highlighted 148 (T0 vs T1), 520 (T0 vs T2) and 135 (T1 vs T2) differentially expressed mRNA-encoding genes **(**Additional file 2: Table S1**)**. Moreover, 85 genes showed DE both in the T0-T1 and T0-T2 comparisons, a result that evidences the high consistency of our results. The analyses of pathways and signalling networks enriched in DE genes with Advaita iPathwayGuide (http://www.advaitabio.com/ipathwayguide) revealed 18 (T0-T1), 18 (T0-T2) and 14 (T1-T2) enriched pathways (Table 1). Similarly, the ReactomeFIViz app identified 34 (T0-T1), 18 (T0-T2) and 15 (T1-T2) pathways (Additional file 3: Table S2). In both analyses, we identified pathways related with (1) T0-T1: circadian clock system, muscle contraction and signaling in cardiomyocytes; (2) T0-T2: circadian rhythm and ribosome pathway; and (3) T1-T2: oxidative phosphorylation, metabolic process and ribosome pathways. Differentially expressed mRNA-encoding genes were also grouped in gene regulatory networks with the ReactomeFIViz app. We found 6 (T0-T1), 20 (T0-T2) and 4 (T1-T2) functional interaction networks which are displayed in Figs. 2, 3 and 4. Several enriched pathways (*q*-value <0.05) such as Wnt signaling pathway (T0-T2), TNF signalling (T0-T1), ATF-2 transcription factor network (T0-T2) and oxidative phosphorylation (T0-T2, T1-T2) are tightly linked to metabolism and energy homeostasis. We also found pathways related with striated muscle contraction (T0-T1) and myogenesis (T0-T2), a result that could be anticipated given the predominance of myofibrilar proteins in the muscle proteome. Other pathways of interest were circadian clock and rhythm (T0-T1, T0-T2), oxidative stress induced gene expression via Nrf2 (T0-T2) and SRP-dependent cotranslational protein targeting to membrane (T1-T2) and eukaryotic translation termination (T1-T2).

**Table 1:** Results of the Advaita Bio’s iPathwayGuide pathway analysis based on the list of genes that are differentially expressed (*q*-value <0.05 and |fold-change| > 1.5) in the porcine *gluteus medius* muscle before (T0) vs 5 h (T1) and 7 h (T2) after eating

T0 vs T1		T0 vs T2		T1 vs T2	
Pathway	*P*-value	Pathway	*P*-value	Pathway	*P*-value
Circadian rhythm	1.00E-03	Ribosome^a^	4.97E-06	Ribosome^a^	2.84E-13
Circadian entrainment	4.00E-03	Circadian rhythm	8.48E-04	Huntington’s disease	2.84E-04
Cholinergic synapse	4.00E-03	Huntington’s disease	1.00E-03	Parkinson’s disease	7.33E-04
Adrenergic signaling in cardiomyocytes	4.00E-03	Legionellosis	5.00E-03	Oxidative phosphorylation^a^	8.74E-04
Transcriptional misregulation in cancer	7.00E-03	Parkinson’s disease	6.00E-03	Alzheimer’s disease	1.00E-03
TGF-β signaling pathway	1.30E-02	Viral myocarditis	7.00E-03	Tight junction	1.30E-02
GABAergic synapse	1.50E-02	Malaria	7.00E-03	Metabolic pathways^a^	1.80E-02
Malaria	1.60E-02	p53 signaling pathway	1.00E-02	Herpes simplex infection	1.80E-02
Cardiac muscle contraction^a^	2.40E-02	Alzheimer’s disease	1.10E-02	p53 signaling pathway	2.50E-02
Herpes simplex infection	2.70E-02	Mineral absorption	1.30E-02	Viral myocarditis	2.90E-02
Fructose and mannose metabolism^a^	3.20E-02	Toxoplasmosis	1.50E-02	Legionellosis	3.20E-02
Neuroactive ligand-receptor interaction	3.20E-02	PPAR signaling pathway	1.90E-02	Amyotrophic lateral sclerosis (ALS)	3.20E-02
Dopaminergic synapse	3.30E-02	Amyotrophic lateral sclerosis (ALS)	2.20E-02	Sulfur metabolism^a^	3.60E-02
Alanine, aspartate and glutamate metabolism^a^	3.50E-02	Sulfur metabolism^a^	2.40E-02	Arrhythmogenic right ventricular cardiomyopathy (ARVC)	5.00E-02
Glutamatergic synapse	3.60E-02	African trypanosomiasis	2.50E-02		
Estrogen signaling pathway	3.70E-02	Transcriptional misregulation in cancer	2.90E-02		
Bladder cancer	4.10E-02	Cardiac muscle contraction^a^	3.30E-02		
Dilated cardiomyopathy	4.90E-02	Tight junction	4.90E-02		

^a^the *P*-value corresponding to the pathway was computed using only over-representation analysis

Considering gene ontology (GO) cellular component, biological process and molecular function related to network functions, the top-scoring networks were (1) T0-T1: *transcription factor complex, circadian regulation of gene expression* and *E-box binding*; (2) T0-T2: *nucleoplasm, negative regulation of transcription from RNA polymerase II promoter* and *structural constituent of ribosome* and (3) T1-T2: *cytosolic small ribosomal subunit, translation* and *structural constituent of ribosome* (Additional file 4: Table S3).

## Discussion

### Post-prandial activation of genes with and without known roles in muscle energy homeostasis

Several of the genes that show the most significant DE between fasted and fed animals (Additional file 2: Table S1) have an established role in metabolism, while for others evidence reported in the literature is more tenuous or even absent. For instance, the 6-phosphofructo-2-kinase/fructose-2,6-biphosphatase 3 (*PFKFB3,* T0-T1: FC = −3.01, *q*-value = 1.91E-07) gene can modulate glucose homeostasis by regulating the levels of fructose-2,6-biphosphate [18], and there are substantial evidences that the G0/G1 switch 2 (*G0S2*, T0-T1: FC = 1.84, *q*-value = 4.03E-02; T0-T2: FC = 2.06, *q*-value = 9.35E-04) protein is involved in the regulation of the rate-limiting lipolytic enzyme adipose triglyceride lipase [19].

The analysis of Additional file 2: Table S1 also evidences the existence of DE for several genes with a plausible but poorly characterized role in metabolism. A good example is the mitoguardin 2 (*MIGA2*, T0-T1: FC = 1.62, *q*-value = 1.86E-02; T0-T2: FC = 2.22, *q*-value = 2.10E-05) gene, which shows a dramatic increase in its expression after food intake i.e. *MIGA2* is 1.62 and 2.22 times more expressed at 5 and 7 h post-ingestion, respectively. This gene encodes a protein that regulates mitochondrial fusion [20]. Noteworthy, mitochondrial dynamics is highly interconnected with the energy status of the cell, and it has been demonstrated that starvation promotes an acute inhibition of mitochondrial fission [21]. Another gene of interest is syndecan 4 (*SDC4*, T0-T1: FC = −1.80, *q*-value = 3.88E-04; T0-T2: FC = −1.82, *q*-value = 9.59E-04), whose expression levels decreased at 5 h and 7 h after ingestion. In mammals, this gene has been mostly related with cell-matrix adhesion, migration, neuronal development, and inflammation, but studies performed in *Drosophila* have revealed that it may also have broad effects on the regulation of energy homeostasis [22]. A third example would be the cysteine- serine-rich nuclear protein 1 (*CSRNP1*, T0-T1: FC = −1.67, *q*-value = 5.37E-03; T0-T2: FC = −1.75, *q*-value = 1.07E-02), a molecule that has been mostly related with T-cell immunity [23] and cephalic neural progenitor proliferation [24]. Interestingly, the expression of this molecule is induced by axin, which appears to promote glucose uptake by enhancing the translocation of GLUT4 [25].

Finally, there is a third category of genes, exemplified by the family with sequence similarity 212, member B (*FAM212B,* T0-T1: FC = 2.04, *q*-value = 3.36E-02; T0-T2: FC = 2.68, *q*-value = 1.13E-06), transmembrane protein 169 (*TMEM169,* T0-T2: FC = 2.83, *q*-value = 6.81E-07) and matrix metallopeptidase 25 (*MMP25,* T0-T2: FC = −2.41, *q*-value = 7.97E-04) loci, that, to the best of our knowledge, have never been reported to participate in the regulation of energy homeostasis.

### The ingestion of food involves changes in the muscle expression of many transcription factors

As shown in Additional file 2: Table S1, we did not detect significant changes in the expression of several genes with a well-established role in lipid uptake (e.g. *CD36*, lipoprotein lipase), synthesis (e.g. acetyl-CoA carboxylase, fatty acid synthase, diacylglycerol O-acyltransferase 1), transportation (e.g. FA binding proteins) and catabolism (e.g. genes of the β-oxidation pathway). One of the few exceptions to this general trend was the lipase G locus (*LIPG,* T0-T1: FC = −1.80, *q*-value = 4.10E-02), which encodes and endothelial lipase modulating lipoprotein metabolism [26]. This gene shows an important drop in its expression levels (1.8 times) 5 h after food intake, a feature that would result in an inhibition of high-density lipoprotein catabolism [26].

We observed DE for many genes encoding transcription factors (Figs. 2 and 3, Additional file 2: Table S1) e.g. the AT-rich interactive domain 5B (*ARID5B,* T0-T2: FC = −2.31, *q*-value = 5.98E-04) gene, which influences adipogenesis and also the accumulation of postnatal lipid storage [27]; Kruppel-like factor 5 (*KLF5,* T0-T2: FC = −1.96, *q*-value = 1.25E-02), that regulates the expression of genes involved in the β-oxidation of FA [28]; *NR4A2*, (T0-T1: FC = −2.16, *q*-value = 8.93E-04), a nuclear orphan receptor that controls the expression of genes related with glucose metabolism [29]; CCAAT/Enhancer Binding Protein δ (*CEBPD,* T0-T1: FC = −2.33, *q*-value = 6.37E-05; T0-T2: FC = −1.84, *q*-value = 1.71E-02) that plays an essential role in adipogenesis [30]; and forkhead box O1 (*FOXO1,* T0-T1: FC = −1.55, *q*-value = 2.12E-02; T0-T2: FC = −1.66, *q*-value = 2.7E-02), which integrates glucose utilization and lipogenesis [31]. In the T0-T2 comparison we found a similar pattern, with DE of genes encoding the nuclear receptor *NR4A3* (FC = −2.28, *q*-value = 1.99E-03), SRY-box 9 (*SOX9,* FC = −2.28, *q*-value = 6.84E-05) and BTB and CNC Homology 1, Basic Leucine Zipper (*BACH2,* FC = −2.45, *q*-value = 4.61E-05) transcription factors, to mention a few (Figs. 2 and 3, Additional file 2: Table S1). In the T0-T2 comparison (Fig. 3, Additional file 2: Table S1), we also detected an increase in the expression levels of the meteorin (*METRNL,* FC = 1.77, *q*-value = 7.33E-03) mRNA that encodes an hormone that promotes energy expenditure and glucose tolerance [32].

### Feeding elicits strong changes in the expression of ribosomal protein genes

Mammalian ribosomes contain 79 different proteins, all of them being encoded by single-copy genes expressed in all tissues [33]. Interestingly, we have detected significant changes in the expression of several ribosomal protein genes (Additional file 2: Table S1). Ribosomal protein genes formed part of the Reactome functional networks shown in Figs. 3 and 4. Moreover, pathways related with ribosomal biogenesis appeared as significant in Table 1 and Additional file 3: Table S2. When nutrients are available, cells tend to activate energy-consuming anabolic pathways whilst under stress or starvation catabolic processes are predominant [33]. Ribosomal biogenesis consumes 60% of cellular energy and this is the key reason why this process is tightly coupled with nutrient supply [34]. The rapamycin (TOR) signalling pathway is deeply involved in coupling ribosome biogenesis with the energy status of the cell by regulating the expression of ribosomal proteins and RNAs [35]. The fundamental role of ribosomal proteins in skeletal muscle metabolism has been illustrated by generating mice where the ribosomal protein S6 cannot be phosphorylated i.e. these mice are viable and fertile but they show muscle weakness and energy deficit [36]. According to our data, these strong changes in the expression of ribosomal protein genes are observed in the T0-T2 and T1-T2 comparisons, but not in T0-T1. Another intriguing observation of our study is that several of these DE ribosomal protein genes are consistently downregulated (e.g. *RPS6KA1, RPL35A, RPS23, RPS21, RPL9* and *RPL39*), a result that is counterintuitive and hard to explain.

### Differential expression of genes related with angiogenesis and oxidative stress

The thrombospondin 1 (*THBS1,* T0-T1: FC = −1.99, *q*-value = 8.00E-03) and 2 (*THBS2,* T0-T2: FC = 2.45, *q*-value = 5.18E-04) and thioredoxin interacting protein (*TXNIP,* T0-T1: FC = −1.78, *q*-value = 1.34E-02; T0-T2: FC = −1.79, *q*-value = 1.13E-02) genes showed significant DE before and after eating (Additional file 2: Table S1). Moreover, they were integrated in the Reactome functional networks depicted in Figs. 2 and 3. These loci have a dual biological role, regulating both angiogenesis and response to oxidative stress. For instance, *THBS1* and *THBS2* are negative regulators of angiogenesis [37, 38] and their expression is down- and upregulated by oxidative stress, respectively [39, 40]. This feature agrees well with our study, since we found a post-prandial (both at T1 and T2) decreased and increased expression of *THBS1* and *THBS2*, respectively. The *TXNIP* protein is one of the main regulators of redox homeostasis [41] and also an angiogenic factor [42]. We have observed a diminished expression of this gene after food ingestion, a finding that agrees well with its function as a promoter of oxidative stress and apoptosis [41].

In the mitochondria, oxidative phosphorylation, by which ATP is synthesized as a source of energy, involves the generation of reactive oxygen species (e.g. superoxide, hydrogen peroxide, hydroxyl radical) as a byproduct [43]. This may promote a state of oxidative stress, i.e. an imbalance between oxidants and antioxidants, resulting in cell and tissue damage. Indeed, a single high-fat meal can temporarily impair endothelial function in healthy individuals and this effect is inhibited by antioxidants [44]. Moreover, lipid peroxidation by reactive oxygen species has been suggested as one of the main mechanisms leading to the development of mitochondrial dysfunction and insulin resistance [45]. On the other hand, it is well known that insulin, which is secreted by the pancreas in response to food ingestion, promotes vasodilation and capillary recruitment in the skeletal muscle, an effect mediated by nitric oxide [46]. These actions on the muscle vasculature are fundamental for the maintenance of glucose homeostasis [47]. As a matter of fact, oxidative stress and neovascularization are two tightly linked biological processes i.e. there are evidences that end products of lipid oxidation can bind the Toll-like receptor 2 promoting an angiogenic response [48]. As a whole, DE of *THBS1*, *THBS2* and *TXNIP* between pre- and post-prandial states probably reflects the combined redox and vascular response of the porcine skeletal muscle to nutrient availability.

### A close relationship between nutritional status and the expression of genes integrated in the muscle circadian clock

One of the main results of our experiment was the detection of DE for a set of genes that form part of the peripheral clock that determines the maintenance of circadian rhythms in the skeletal muscle (Figs. 2 and 3, and Additional file 2: Tables S1, Additional file 3: Tables S2 and Additional file 4: Tables S3). Patterns of DE in the two available comparisons (T0-T1 and T0-T2) were consistent i.e. there was an upregulation of *ARNTL* (T0-T1: FC = 1.87, *q*-value = 193E-0.4; T0-T2: FC = 2.43, *q*-value = 2.99E-13) and *NR1D1* (T0-T1: FC = 1.61, *q*-value = 8.30E-03; T0-T2: FC = 1.87, *q*-value = 9.52E-04), and a downregulation of *PER1* (T0-T1: FC = −2.85, *q*-value = 3.95E-11; T0-T2: FC = −1.83, *q*-value = 1.12E-0.2), *PER2* (T0-T1: FC = −1.67, *q*-value = 4.33E-04, T0-T2: FC = −2.48, *q*-value = 7.03E-14), *BHLHE40* (T0-T2: FC = −1.77, *q*-value = 7.87E-0.5), *SIK1* (T0-T1: FC = −2.62, *q*-value = 1.91E-07), *CIART* (T0-T1: FC = −2.16, *q*-value = 5.79E-05; T0-T2: FC = −2.35, *q*-value = 4.52E-06) and *CRY2* (T0-T2: FC = −1.60, *q*-value = 1.28E-0.2). In mammals, the circadian clock is regulated by either the CLOCK-ARNTL or the NPAS2-ARNTL heterodimers depending on the tissue under consideration [49]. These heterodimers activate the transcription of the Period (*PER1* and *PER2*) and Cryptochrome (*CRY1* and *CRY2*) genes [49]. In diurnal species, the PER and CRY complexes accumulate in the cytoplasm during daytime and they are translocated to the nucleus in the evening, thus repressing their own expression through the interaction with CLOCK/ARNTL [49]. The BHLHE40 molecule is a negative regulator of the ARNTL-CLOCK complex [50]. Other clock genes of interest are *SIK1*, that regulates the entrainment of the circadian clock [51], *CIART*, whose inactivation increases the circadian period of locomotor activity in mice [52] and *NR1D1*, a critical regulator of the circadian clock with strong effects on lipid homeostasis [53].

Our data indicate that food ingestion modulates the expression of circadian genes in the porcine skeletal muscle. It might be argued that this DE is just the obvious consequence of slaughtering pigs at different timepoints (T0 = 0 h., T1 = + 5 h. and T2 = + 7 h.). However, studies performed in model species have revealed that the feeding/fasting cycle is one of the main zeitgebers (time cues) synchronizing the skeletal muscle clock [54]. Noteworthy, this clock plays a key role in muscle physiology by regulating the expression of more than one thousand genes mainly involved in metabolic processes [55]. Muscle lipid deposition in pigs could be affected by the expression of these genes because their inactivation in mouse has evidenced numerous metabolic abnormalities including ectopic fat in the muscle, reduced circulating levels of triglycerides and free fatty acids, obesity, hyperlipidemia and severe hepatic steatosis [49]. Besides, SNPs in the human clock genes have been related with abdominal obesity, increase in carbohydrate intake, higher body mass index and metabolic syndrome [56].

## Conclusions

Our results indicate that the ingestion of food affects the expression of many transcription factors that are essential for coordinating the metabolic response triggered by the availability of nutrients. Amongst these, clock genes could be particularly important due to their key role in the adequate synchronization of this response as well as because of their broad effects on muscle metabolism. We have also shown that several genes without an evident link with muscle metabolism change their expression in response to nutrient inflow, an observation that suggests that our knowledge about the genetic basis of energy homeostasis in the porcine muscle is still quite limited. Given the close physiological similarity between pigs and humans, data presented in the current study could be also of interest to understand the consequences of food intake on gene expression in this latter species.

## Additional files

Additional file 1: Figure S1. Kinetics of triglyceride and non-esterified fatty acids (FA) concentrations in 36 Duroc pigs at three time points: before eating and 5 and 7 h post-ingestion. (GIF 17 kb)

Additional file 2: Table S1. Differentially expressed genes (*q*-value <0.05 and |fold-change| > 1.5) in the pig gluteus medius muscle at fasting (T0) vs 5 h (T1) vs 7 h (T2) after eating. (XLSX 900 kb)

Additional file 3: Table S2. Pathways identified by ReactomeFIViz as enriched in differentially expressed genes (*q*-value <0.05 and |fold-change| > 1 .5). Three conditions were compared: fasting (T0), 5 h after eating (T1) and 7 h after eating (T2). (XLSX 13 kb)

Additional file 4: Table S3 Gene regulatory networks identified with the ReactomeFIViz app, considering GO biological process, molecular function and cellular component (*q*-v﻿alue<0.05). (XLSX 50 kb)
