# Supplementary figures and images for: Nutrient supply affects the mRNA expression profile of the porcine skeletal muscle
Source: BMC Genomics. 2017 Aug 10;18:603. doi: 10.1186/s12864-017-3986-x (PMC5553784; doi:10.1186/s12864-017-3986-x)

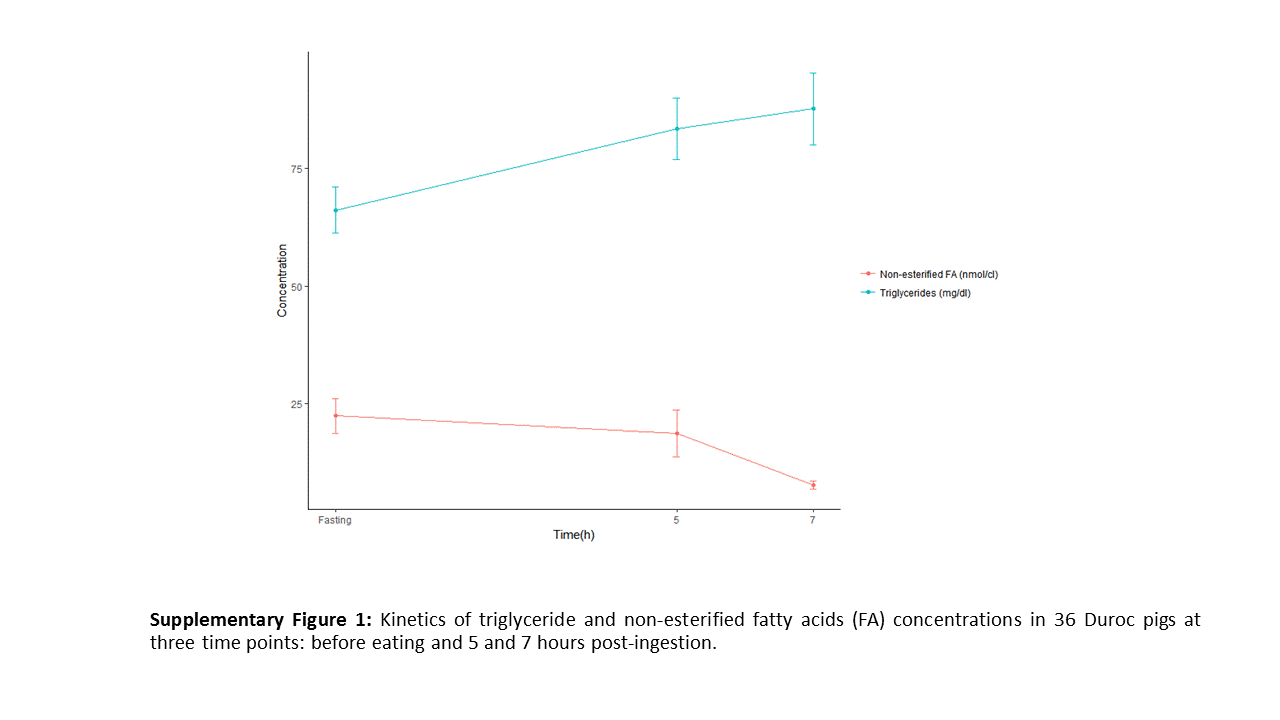

Supplement: Supplementary file 1 — Kinetics of triglyceride and non-esterified fatty acids (FA) concentrations in 36 Duroc pigs at three time points: before eating and 5 and 7 h post-ingestion. (GIF 17 kb) [file 12864_2017_3986_MOESM1_ESM.gif]
